# Experimental Study on Instability of Shotcrete Reinforced Slope Based on Embedded Anchor Sensor

**DOI:** 10.3390/s25206493

**Published:** 2025-10-21

**Authors:** Hai Ning, Junkai Ou, Jihuan Jin

**Affiliations:** Department of Civil Engineering, Yanbian University, 977 Park Road, Yanji 133002, China

**Keywords:** embedded sensing, GFRP, slope collapse, early warning, shotcrete reinforcement

## Abstract

Given the limitation of existing slope collapse monitoring technology, which relies on surface sensors, and the difficulty in capturing the precursors of deep rock and soil instability, this study used rock anchor embedded sensing technology to conduct collapse tests on artificial simulated slopes. Two groups of control conditions were designed: (1) shotcrete reinforced slope and natural slope; and (2) GFRP anchor and spiral steel anchor support system. The deformation characteristics of the slope at the initial stage of collapse were analyzed. The results show that the monitoring method based on the stress–strain response of deep rock mass significantly improved the early warning effect. GFRP anchor had a lower elastic modulus and responded more sensitively to small displacements than spiral steel anchor. Shotcrete reinforcement transformed slope deformation from ‘local dispersed deformation’ to ‘overall coordinated deformation’ and delayed slope instability via the ‘deformation hysteresis effect’. This study provides key technical parameters for the intelligent monitoring system of high-risk slopes as well as support for pre-disaster emergency evacuation decision-making and the establishment of intelligent early warning systems.

## 1. Introduction

As an active support measure, rock bolts can effectively limit rock deformation and control the expansion of potential cracks and are widely used in mining, transportation, water conservancy, and other engineering projects [1,2]. However, engineering practice shows that even rock masses reinforced with anchor bolts may still suffer varying degrees of instability and damage under the effects of earthquake loads, continuous weathering, groundwater erosion, or construction quality defects, and may even cause serious secondary disasters such as landslides and collapses [3].

Most rock anchor reinforcement systems currently in widespread use are traditional mechanical structures. While these systems exhibit good load-bearing performance during initial support, they generally lack built-in sensing and information feedback capabilities, which makes it difficult to real-time monitor stress changes, deformation development, and the synergistic state of the surrounding rock mass throughout their service life. Once an abnormality occurs inside the rock mass or the bearing performance of the anchor is degraded, it is often impossible to detect it in time due to the lack of effective monitoring, thus missing the best time for early warning and intervention [4]. This “disconnection between support and monitoring” has become one of the technical shortcomings in current rock mass engineering safety management. In response to this problem, many scholars at home and abroad have conducted extensive research and exploration on the rock anchor reinforcement mechanism, response characteristics, and monitoring technology. Laboratory experiments have confirmed that the increase in interlayer cohesion and the greater number of rock bolts can significantly improve the anchoring performance of layered rock masses [5]. Li et al. (2022) [6] suggested that the mechanical characteristics of rock bolts under single-layer and multi-layer bedding separation be studied through numerical simulations, revealing the correlation between bolt length and additional stress. Yu et al. (2024) [7] proposed the rock anchor preload loss effect theory, pointing out that the stress attenuation of deep rock anchors would affect the monitoring accuracy. Mesutoglu and Ozkan (2024) [8] compared the equivalent support effects of rock anchors and steel supports but did not involve sensing. Wang et al. (2025) [9] demonstrated through discrete element simulation that increasing the bolt density could significantly enhance the reinforcement effect. In recent years, integrating sensing technology into the anchor to achieve the in situ and real-time monitoring of the internal deformation of the rock mass and the stress state of the anchor has become a research hotspot, for example, fiber optic sensing technology [10,11], piezoelectric ceramic sensors [12], resistance strain gauges [13,14], etc. Although these studies have laid the foundation for the development of smart anchors, their deformation monitoring performance and early warning effectiveness in complex slope systems still need to be further verified in control model tests.

Most existing studies have focused on the mechanical performance testing of individual rock bolts, with little consideration given to their synergistic effect with “shotcrete surface reinforcement” in practical engineering. Shotcrete is a commonly used protective measure for rocky slopes, but the mechanism of deep deformation monitoring under the synergy of these two components remains unclear. Most existing tests were conducted on small-scale components and failed to compare the response differences of anchors under simulated real slope geometry and excavation conditions. Therefore, it is necessary to rely on controlled model tests to build a coordinated monitoring system for anchors and shotcrete under real working conditions, explore the deformation characteristics of deep rock masses, and fill the gap between current experiments and actual engineering applications.

Traditional spiral steel anchors are widely used in engineering practice due to their high strength and mature construction technology [15]. However, this type of anchor has significant shortcomings: (1) the material is easily corroded, has high density, and has poor long-term durability, especially in moist, corrosive or weathered environments, where the service life is difficult to guarantee [16]; and (2) more importantly, its stiffness and strength are extremely high, resulting in very weak measurable strain signals generated by the anchor itself when the rock mass undergoes slight displacement. This makes it difficult to effectively capture these critical early slight deformation signals even if high-precision sensors are integrated, limiting its sensitivity as a “sensing element”. In contrast, the glass fiber reinforced composite (GFRP) anchors that have emerged in recent years have significant advantages such as high specific strength, good corrosion resistance, light weight, and strong insulation [17], and are particularly suitable for geotechnical environments with high humidity, high corrosion or electromagnetic interference sensitivity [18,19]. In addition, the elastic modulus of GFRP material is typically 0.2 times that of steel, which means that under the same external load or rock displacement, GFRP anchors will produce larger measurable strains, significantly improving the sensitivity to small deformations. Furthermore, GFRP materials are highly designable and easy to integrate with sensor elements, which helps to build smart anchor rod structures with self-sensing capabilities [20,21,22]. This advantage in material properties gives it great potential in capturing early weak signals of deep rock instability.

However, while GFRP anchors have demonstrated promising performance in theoretical analysis and small-scale component testing, their overall reinforcement effectiveness and instability response characteristics under complex working conditions remain poorly characterized. He et al. (2021) [23] compared the bond damage characteristics of steel and GFRP anchors in reinforcing soft rock slopes, but did not conduct tests on their sensing performance under small deformations. Bai et al. (2024) [24] studied the bonding performance of the grout–rock interface of GFRP bolts through field tests and found that the bonding strength reached 1.2 MPa. However, this study did not involve the monitoring of the synergistic deformation between the shotcrete and bolts. Ma et al. (2025) [25] found through indoor tests that the fracture of GFRP anchors mostly occurred in the middle of the anchor rod body, and that the interfacial bonding strength increased linearly with the increase in glass fiber content. This study clarified the mechanical failure mechanism of GFRP anchor rods, but did not combine mechanical properties with sensing functions. Sun et al. (2023) [26] analyzed the synergistic anchoring effect of GFRP anchors and concrete through numerical simulation, and pointed out that the lower elastic modulus of GFRP could reduce the interfacial stress concentration. However, this simulation neither accounted for the strain transfer law in real slopes nor verified the feasibility of using GFRP as a sensing element. Existing research has primarily focused on testing the mechanical properties of individual anchors, with limited consideration of their synergistic effects with real-world reinforcement systems such as natural slopes, jointed rock masses, or shotcrete. In particular, systematic experimental studies of GFRP anchors under controlled model testing conditions are lacking, making it difficult to fully understand their response mechanisms during slope instability and their monitoring and early warning capabilities.

In addition, in the existing slope model tests, few studies have comprehensively considered the surface reinforcement effect of shotcrete and the deep support performance of rock anchors. Rahayu et al. (2024) [27], through rainfall simulation tests, verified that a shotcrete layer prepared from concrete waste could improve the anti-sliding stability of the slope by 25% to 30%. However, this study only judged the stability by surface displacement monitoring and did not conduct an in-depth analysis of the coordination mechanism of deep rock deformation. Bian et al. (2024) [28] carried out a physical model test of double-layer slope instability caused by rainfall and found that a 10–15 mm thick shotcrete layer could delay the slope failure time by 60–90 s. However, their study did not include anchor monitoring data, so it was impossible to identify the deep dynamic characteristics of this hysteresis effect. In Wang et al. (2022) [29], although their study revealed the macro-reinforcement effect of shotcrete, they failed to establish the correlation chain of “surface reinforcement–deep deformation–monitoring signal”, and their research results could not directly support the parameter design of the intelligent monitoring system.

To address the increasing frequency of slope instability caused by extreme rainfall and earthquakes and the urgent need for intelligent adaptive reinforcement measures [30,31], this study combined embedded sensing technology for fiberglass anchors with shotcrete reinforcement in an artificially simulated slope system. Two control conditions, “shotcrete reinforcement (Case 1)/no reinforcement (Case 2)” and “GFRP/spiral steel anchor”, were set to monitor deep deformation under the synergistic effect of the “anchor-reinforcement system”. By simulating the instability process of layered slope excavation, this study quantitatively compared the deformation responses of glass fiber reinforced plastic (GFRP) and steel anchors. The study explored the synergistic effect of surface shotcrete reinforcement and deep anchor support during slope deformation as well as the strain transfer behavior of GFRP anchors during deep rock microdeformation. Discrete element analysis was used to verify the sensitivity of the GFRP anchors in detecting deep microdeformations. This research not only bridges the gap between small-scale GFRP anchor testing and practical engineering applications, but also provides a new technical approach for improving the early warning accuracy and disaster prevention capabilities of slope engineering projects under extreme conditions such as heavy rainfall and earthquakes.

## 2. Materials and Methods

### 2.1. Introduction to Rock Slope Model

**Figure 1:**
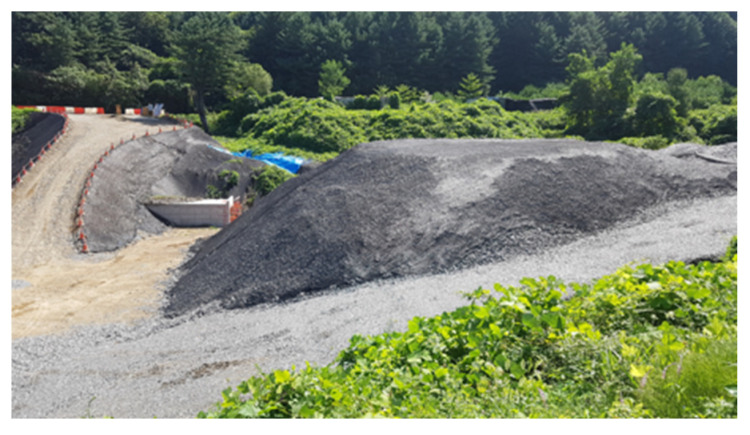
Construction of the model slope.

To verify the actual monitoring performance of rock bolt sensors, the ideal method is to install them in situ on a real rock slope and conduct collapse tests. However, due to site conditions and safety considerations, destructive testing on natural slopes is difficult to implement. Therefore, this study innovatively used a rock pile site generated by tunnel construction as an experimental platform to construct an artificial simulated slope to test the sensor’s performance (Figure 1).

**Figure 2:**
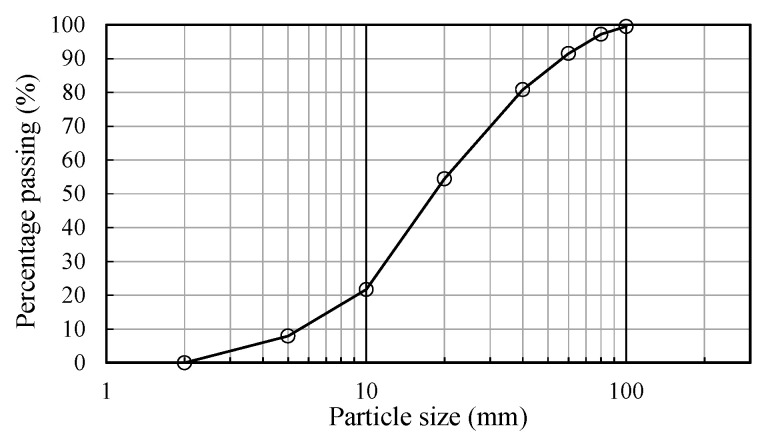
Particle size curve of the slope material.

The tunnel construction waste rubble used in this study, though artificially deposited, exhibited mechanical properties that are representative of natural slope debris [32]. Figure 2 shows the particle size curve of the rubble, and Table 1 presents its mechanical parameters. For the experiments, layered stacking was combined with excavator bucket compaction, resulting in a compaction degree of 85–90%—consistent with the compaction state of loose, crushed surface areas in natural slopes (80–95%). This approach further minimizes the discrepancy between the prepared materials and natural conditions. Artificial slopes cannot fully replicate the complex environment of natural slopes, but parameter matching ensures their rationality within the core research objective of “crushed body mechanical response”, laying the foundation for subsequent field verification.

After compaction and stacking by an excavator, a standardized test slope with a slope angle of 56° and a crest height of 8.0 m was formed. The slope angle of 56° corresponded to the common slope angle of typical high and steep slopes [33]; this ensured a high degree of correlation between the experimental conditions and the actual engineering requirements.

Such an artificial slope model not only provides a safer and more controllable testing environment than natural slopes, but also allows for repeated loading and unloading cycles to be carried out under standardized conditions. The progressive excavation method adopted in this study has been widely recognized in geotechnical model testing as it enables a gradual reduction in the resisting mass and thus effectively reproduces the stepwise weakening process leading to slope collapse [34]. Furthermore, by combining anchor-embedded sensing data with complementary monitoring methods, this integration of multi-source monitoring significantly enhances the reliability of the experimental results and provides a robust basis for evaluating the early warning potential of different anchor sensor systems.

### 2.2. GFRP and Steel Anchor Sensor Manufacturing Method

**Figure 3:**
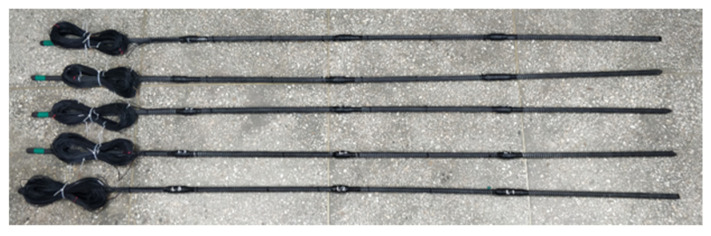
GFRP anchor strain sensor.

**Figure 4:**
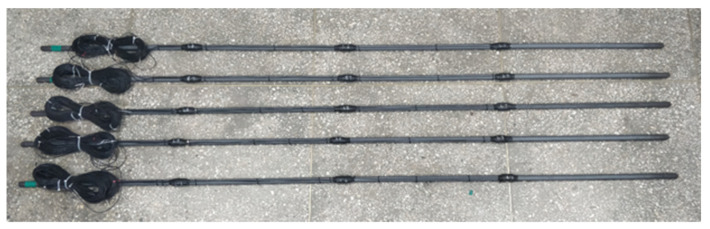
Steel anchor strain sensor.

This study used GFRP and spiral steel anchors to fabricate rock bolt sensors. During fabrication, a 25 mm outer diameter, 3000 mm long rock bolt was divided into four equal parts. A 100 mm × 10 mm rectangular smooth area was polished on the surface for sensor mounting. Figure 3 and Figure 4 show the appearance of the sensors. The sensors were arranged in a linear pattern and wrapped with insulating tape for protection, waterproofing, and insulation. Finally, all sensors were coated to prevent breakage during installation. Table 2 provides the technical parameters of GFRP anchors.

**Figure 5:**
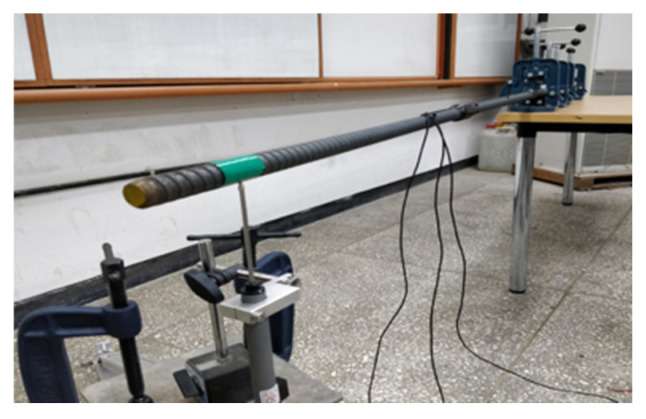
Sensor strain–displacement relationship measurement.

**Figure 6:**
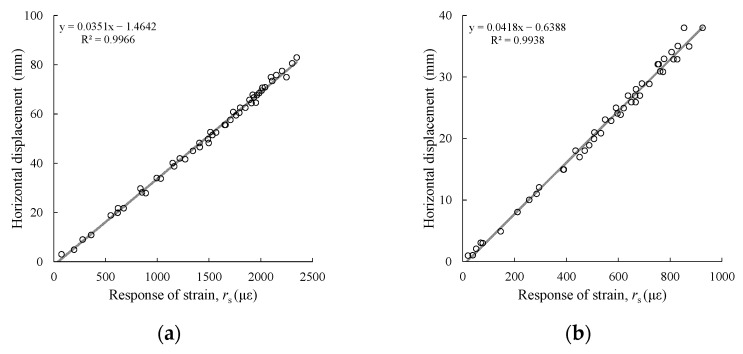
Displacement–strain relationship of the strain sensors: (**a**) GFRP anchor; (**b**) steel anchor.

**Figure 7:**
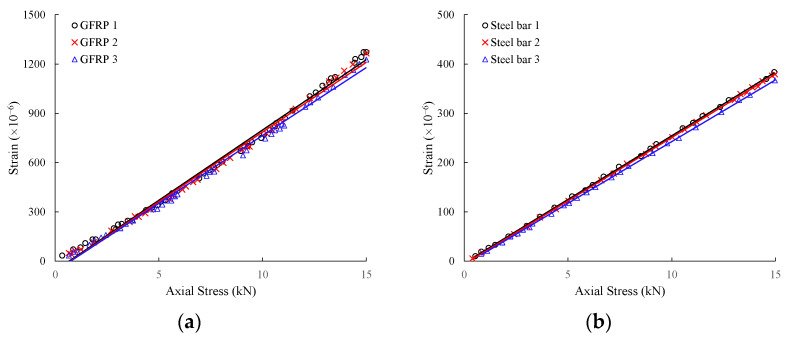
Repeatable calibration curves for the GFRP/steel anchor sensors: (**a**) GFRP anchor; (**b**) steel anchor.

As shown in Figure 5, this study used the cantilever beam calibration method to verify the performance of the two displacement sensors. The specimen was fixed at the right quarter end, and a load was applied to the free end. The following measurements were made: (1) the relationship between displacement and response strain was measured; and (2) the coefficient of variation of the strain values was measured. The calibration results for displacement and response strain are shown in Figure 6, where “strain” represents the axial deformation rate of the anchor bolt, and “displacement” represents the change in slope or anchor bolt position. Both sensors exhibited a good linear relationship between displacement and strain. The strain response of the free end of the anchor rod under load showed high discrimination. Furthermore, the coefficients of variation of the strain measurements under the same load for three anchor rod sensors produced in the same batch were 1.4% to 4.0% for the GFRP anchors and 0.8% to 3.9% for the steel anchors, respectively. Figure 7 shows that the inherent accuracy of the sensor was stable, reducing the discreteness of the results due to sensor errors.

To clarify the “quantitative indicators and analytical basis for early warning”, reference was made to the relevant provisions of Bai et al. [24]. The strain monitoring standard was set at 30% of the anchor’s ultimate strain, which was 1.42% for the GFRP anchor and 0.15% for the spiral steel anchor (Table 3). The early warning time was defined as the interval between the first sudden change in the sensor’s real-time monitoring value and the occurrence of slope instability. Strain sensitivity was used to quantify the response of the two anchor types to small deformations.

### 2.3. Anchor Sensor Layout

**Figure 8:**
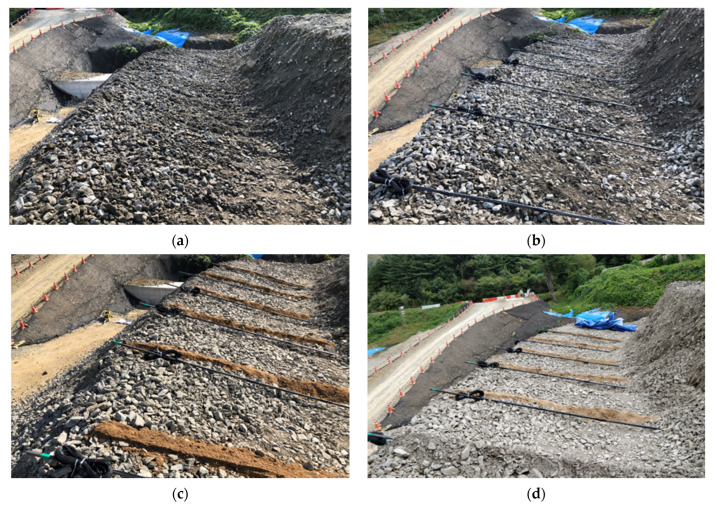
Sensor implantation in the model slope. (**a**) Excavation of the upper rock layer. (**b**) Alignment of sensors. (**c**) Laying of a 20 mm thick sand layer. (**d**) Covering with a rock layer.

This study used a top-mounted data cable connected to a data logger to automatically collect and record sensor data. The specific implementation process is shown in Figure 8: First, the slope was excavated 1.5 m below the top of the slope, and the GFRP and spiral-reinforced rock anchor sensors were symmetrically arranged every 1.5 m to effectively cover the potential sliding surface and realize distributed monitoring of the slope deformation field. A 20 mm thick sand layer was then laid as a base, completing the sensor installation. The area surrounding the sensors was then cast with cement mortar at a water–cement ratio of 1:3 to simulate real-world anchoring conditions. Gravel was then evenly covered and backfilled.

## 3. Slope Collapse Test

**Figure 9:**
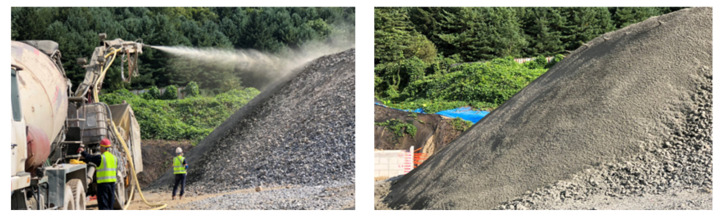
Shotcrete operation process and completion scene.

This study implemented shotcrete reinforcement on the rockfill slope to more realistically simulate the characteristics of rock slopes. Figure 9 shows the construction site of shotcrete with a thickness of 10 mm, designated as Case 1. The untreated gravel slope was designated as Case 2. The shotcrete pressure was 0.4 MPa, the selected thickness was 10 mm, and the compressive strength was approximately 15 MPa (7 days), representing the lower limit for typical engineering applications. This ensured that the influence of surface reinforcement could be isolated without significantly altering the global mechanical characteristics of the rockfill slope. By contrasting the reinforced and unreinforced cases, the experiment allowed for a systematic evaluation of the role of shotcrete in delaying crack initiation, modifying deformation evolution, and mitigating large-scale collapse.

**Figure 10:**
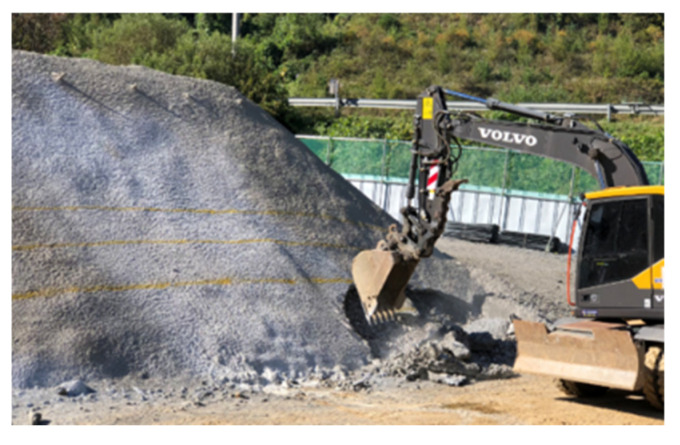
Excavator excavating slope.

This study simulated the slope instability process using a staged excavation method, starting at the toe of the slope and observing the collapse behavior of the model slope. Figure 10 shows a two-stage excavation using a backhoe: the first stage excavation depth was 3.0 m, and the second stage excavation depth was 4.0 m. The verticality of the excavation surface was strictly controlled during the construction process. Although a 2.0 m test excavation was conducted in the initial stage, the monitoring data did not reach a significant level of change and was not used in subsequent analysis. Furthermore, to ensure comparability of the test data, a dedicated data collection waiting period was implemented after each excavation stage to fully understand the slope deformation characteristics while strictly controlling the excavation depth.

## 4. Results

### 4.1. Analysis of Experimental Results

**Figure 11:**
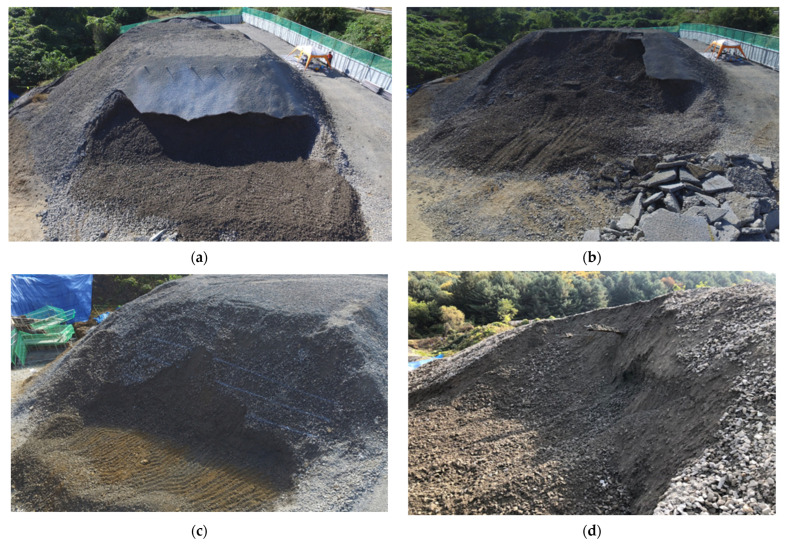
Excavation model slopes. (**a**) Shotcrete reinforced slope excavation 3 m. (**b**) Shotcrete reinforced slope excavation 4 m. (**c**) Unreinforced slope excavation 3 m. (**d**) Unreinforced slope excavation 4 m.

Figure 11a,b illustrates the test scenarios of an 8-m-high slope excavated to 3 and 4 m depth, respectively, after shotcreting (Case 1). Figure 11a shows the irregular, jagged shape of the excavation surface due to the impact of the backhoe excavation, with some areas even extending upward beyond the intended excavation line. Figure 11b shows a panoramic view of the slope collapse after excavation. The collapse of the rock mass below the excavation line triggered sliding of the rock mass beneath the shotcrete layer, resulting in the collapse of a large area of the concrete layer.

Figure 11c,d shows the test process and results for an 8-m-high fill slope (Case 2) at excavation depths of 3 and 4 m. Figure 11c shows the localized upwelling and sliding of rock blocks during the 3 m vertical excavation. Figure 11d shows that after the 4 m excavation, the upper portion of the slope completely collapsed, exposing the rock bolt sensors.

**Figure 12:**
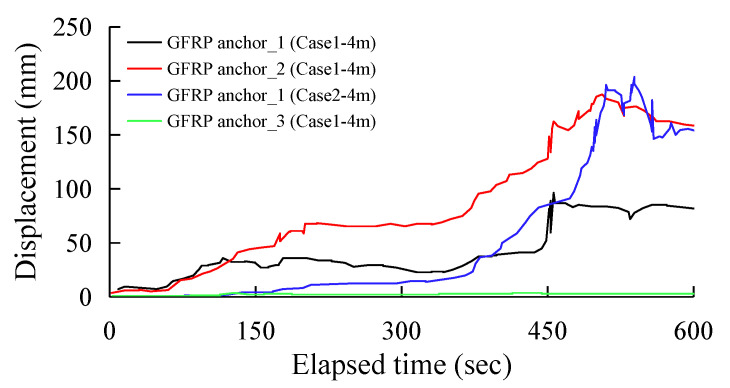
All GFRP anchor displacement data during a 3.0 m height excavation.

**Figure 13:**
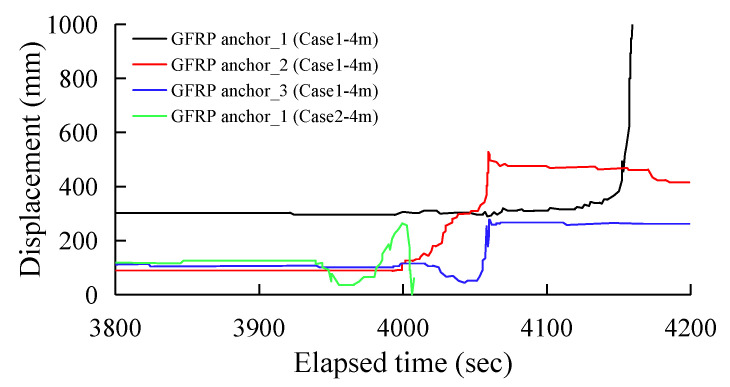
All GFRP anchor displacement data during a 4.0 m height.

Figure 12 and Figure 13 show the displacement distribution of the GFRP anchor sensors over excavation time under the two experimental conditions, Case 1 and Case 2, respectively. Figure 12 records three sets of GFRP anchor sensor data on the shotcrete slope during the 3 m excavation phase and one set of data on the rockfill slope. After 540 s of excavation, the maximum displacement of the shotcrete slope reached 96 mm to 203 mm, while the rockfill slope only experienced a displacement of approximately 3 mm. When the toe of the slope was excavated 3 m, the support force at the bottom of the slope weakened. The shotcrete layer then caused the underlying rock mass to deform synergistically, resulting in an overall sliding trend. Because the GFRP anchor was embedded deep in the rock mass and interacts with the shotcrete–rock structure, its displacement directly reflects this overall deformation. Case 2 involves an untreated rubble slope. The loose rock mass lacks overall restraint and relies solely on intergranular friction for stability. Excavation to a depth of 3 m had not yet reached the critical threshold for overall slope instability. Only minor upwelling or sliding of localized rock blocks due to loss of support occurred, without causing widespread deformation. It can be seen that even during the initial deformation phase of 3 m of excavation, the slope reinforced with shotcrete exhibited distinct deformation characteristics from the unreinforced slope.

Figure 13 shows four sets of monitoring data from the 4 m excavation phase. Calculated at 3800 s after the start of the second phase of excavation, all members except GFRP anchor_1 had stabilized. GFRP anchor_1, located at the edge, maintained a displacement of approximately 300 mm due to the deadweight of the damaged shotcrete blocks. Overall sliding in the shotcrete-reinforced slope is triggered by shear failure in the deep rock mass and is a form of plastic deformation. Staged excavation continuously weakens the slope’s anti-slip force. When deformation reaches the critical value, the synergistic effect between the concrete layer and the rock mass fails, leading to overall collapse. Since plastic deformation is irreversible, the displacement recorded by the GFRP anchors continues to increase with no rebound.

**Figure 14:**
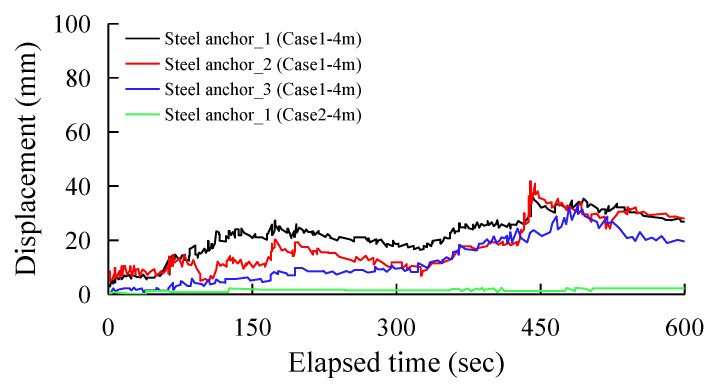
All steel anchor displacement data during the 3.0 m height excavation.

**Figure 15:**
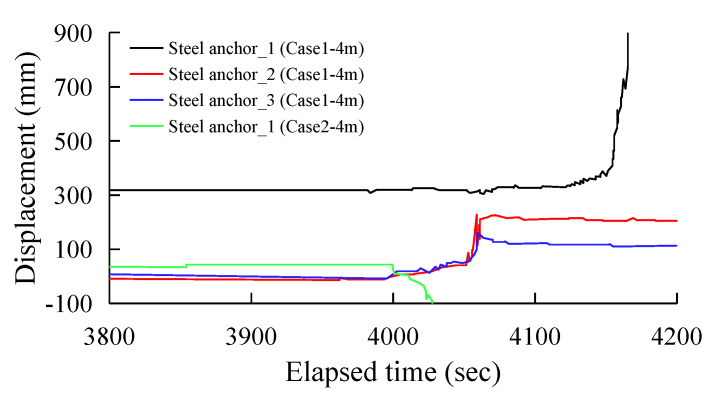
All steel anchor displacement data during the 4.0 m height excavation.

Figure 14 and Figure 15 show the displacement distribution of the spiral steel anchor sensors over excavation time for Scheme 1 and Scheme 2, respectively. Figure 14 records the monitoring data from three sets of spiral steel anchor sensors on the shotcrete slope and one set of sensors on the fill slope during the 3 m excavation phase. The data shows that after 400 s of excavation, the maximum displacement of the shotcrete slope reached 36–42 mm, while the displacement of the fill slope was only 2 mm. Similar to the GFRP anchor sensors, the spiral steel anchors also produced significant displacement on the shotcrete slope. Figure 15 shows the monitoring records from four sets of sensors during the 4 m excavation phase. Taking the start time of the second phase of excavation at 3800 s as the baseline, the spiral steel anchor sensors on the rock fill slope recorded an 80 mm displacement at 200 s, followed by a sudden drop. The shotcrete slope began to show significant movement after 260 s of excavation, with the maximum displacements of steel anchor_2 and steel anchor _3 reaching 190 mm and 140 mm, respectively. The displacement of steel anchor_1 exceeded the limit threshold.

When the excavation depth reached 4 m, the support force at the slope toe weakened significantly and fell below the critical value required to maintain the overall slope stability. Consequently, the upper portion of the unreinforced slope collapsed completely, and the anchor sensors were exposed as the surrounding rock mass lost its restraining effect. The displacement monitoring signal was interrupted, and the displacement value dropped sharply, consistent with the characteristics of the instantaneous collapse of loose rock mass under critical load. On the other hand, the shotcrete-treated slope, because the concrete layer cements the loose gravel on the surface into a single entity, thus strengthening the slope’s integrity, its bond with the rock mass and its own tensile strength created a temporary resistance. Significant displacement occurred later than on the unreinforced slope, indicating a delayed overall instability. There was also a time lag between excavation and overall instability, manifesting as a deformation hysteresis.

**Figure 16:**
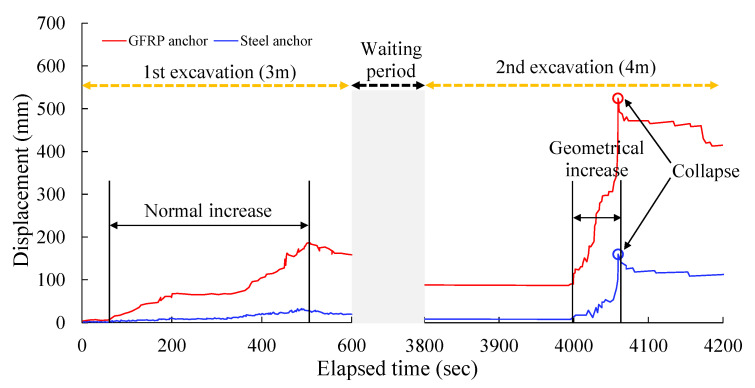
Displacement changes of the GFRP and steel anchors at different stages.

Figure 16 shows the displacement changes during the stable increase and accelerated increase stages measured by the ground displacement meter. During the first excavation stage with a depth of 3 m, the displacement of the GFRP anchor showed a “normal increase” and gradually increased over time. In contrast, the displacement of the steel anchors increased very slowly and remained almost stable, which shows that the GFRP material responded more significantly to the stress release of the rock and soil mass in the early stages of excavation and could reflect the displacement changes of the rock and soil mass earlier. During the non-excavation waiting period, the displacement of the GFRP anchor fluctuated slightly and then tended to be relatively stable, while the displacement of the steel anchors still did not change much. This proves that although GFRP accumulated more displacement in the early stage, both could maintain a relatively stable state without excavation disturbance.

In the second excavation stage, when the depth was 4 m, the displacement of the GFRP anchor showed “geometric growth”, rapidly rose to a peak, and then “collapsed” as the displacement decreased, indicating that the rock and soil mass where the GFRP anchor is located may lose stability due to excessive deformation and cause damage. Although the displacement of the steel anchors increased during this excavation, the amplitude was much smaller than that of the GFRP anchors, and there was no sudden collapse-like displacement decrease. This shows that the steel anchors can control deformation relatively well and maintain structural stability when subjected to large excavation disturbances.

In summary, it can be seen from the comparison of the curves that the GFRP anchor rod is more sensitive to displacement response and has larger deformation during the excavation process. Especially during the second deep foundation pit excavation, it is easy to cause rock and soil collapse due to excessive deformation. However, the displacement changes of the steel anchors were small, the mechanical stability was strong, and it could better resist the displacement disturbance of rock and soil caused by excavation.

As shown in Table 4, during the two excavation stages (3.0 m and 4.0 m), the early warning performance of the two anchors exhibited distinct regular differences, with the GFRP anchors consistently demonstrating superior early warning capabilities. In the 3.0 m excavation stage, the time for the GFRP anchor to reach the early warning threshold for the first time was 391 s, which was 13 s earlier than the 404 s of the spiral steel anchor; the corresponding early warning time was 114 s, which was 1.28 times that of the spiral steel anchor (89 s). The corresponding displacement of the slope monitored by the two was significantly different, the GFRP anchor was 90 mm, and the spiral steel anchor was 13 mm. It can be seen that in the earlier displacement stage, the GFRP anchor could trigger an early warning, and the capture of slope deformation as more forward-looking. In terms of strain sensitivity, the GFRP anchor rod had a strain sensitivity of 28.4 με/mm at the 3.0 m excavation stage, an 18.3% increase compared with the spiral steel anchor rod (24.0 με/mm). This further demonstrates that it has a stronger ability to capture small slope deformations and can perceive changes in the internal stress state of the rock mass earlier.

Entering the 4.0 m excavation stage, the difference in early warning performance of the two anchors further diverged, and the early warning advantage of GFRP anchors became more prominent. The time it took for the GFRP anchor to reach the early warning threshold for the first time was 4011 s, which is basically the same as that of the spiral steel anchor (4016 s), with a difference of only 5 s; however, the corresponding early warning time was 48 s, 5 s longer than the spiral steel anchor (43 s), the corresponding displacement gap of the slope was significantly expanded at this time, and both exceeded the warning threshold, 397 mm for the GFRP anchor and 143 mm for the spiral steel anchor, indicating that during the large deformation stage of the slope, the GFRP anchors could continuously provide an early warning window within a larger displacement range, leaving more time for emergency response. In terms of strain sensitivity, the numerical difference between the GFRP anchors and spiral steel anchors was consistent with the 3.0 m stage. The stability of the GFRP anchors is more in line with the needs of long-term monitoring and early warning, and does not affect its overall performance superiority.

### 4.2. Discrete Element Analysis

This study used the three-dimensional discontinuity analysis program 3DEC to investigate the behavior of the ground following excavation of a crushed rock slope at a tunnel construction site.

**Figure 17:**
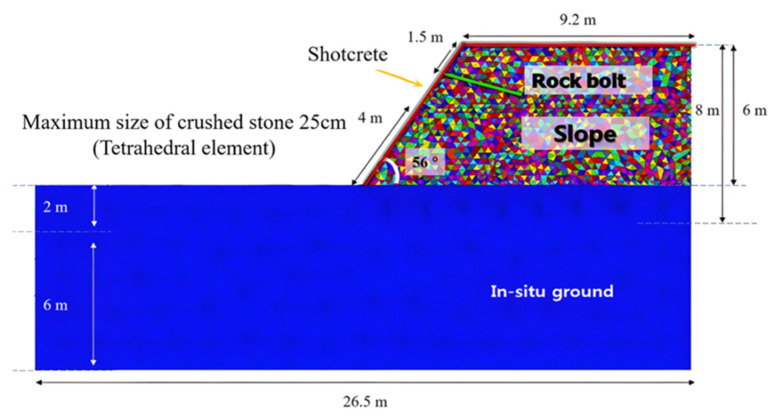
Discrete element analysis model.

The discrete element method (DIM) accounts for the characteristics of the discontinuity by modeling the structure as a single block. To account for the contact behavior of the discontinuity, the method utilizes the stiffness, shear, and tension properties of the contact surface. The explicit finite difference method (FDM) was used to numerically integrate the equations of motion. Figure 17 shows the model used for the analysis. The crushed rock was modeled as a single tetrahedral block, the underlying ground consisted of a continuum, the anchor bolts (3 m long) were modeled using cable elements, and the shotcrete was modeled using lining elements.

**Figure 18:**
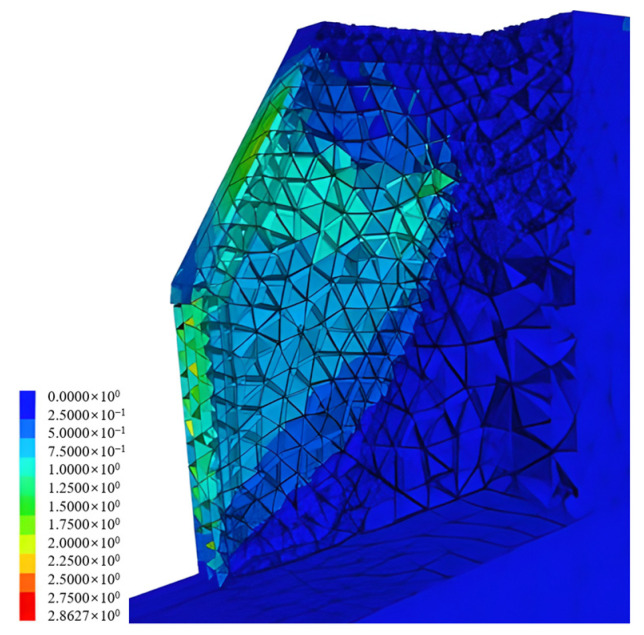
Distribution trend of ground displacement (mm) after excavation.

This analysis considered the contact characteristics between rock boulders. Considering that the boulders were embedded in the original foundation, the slope model properties were defined in two ways. The cohesion of the original foundation was estimated to be zero or very low, but due to the presence of interlocking rock boulders, the cohesion was taken as 0.3 MPa. Figure 18 shows the surface displacement distribution after excavation of the rock bolt-embedded slope. After excavation, large displacements were observed in the slope front and in the rock bolt-embedded area.

**Figure 19:**
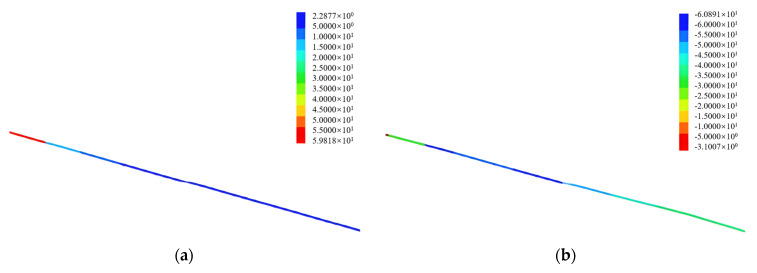
Rock bolt displacement and axial force distribution: (**a**) displacement (mm); (**b**) axial force (kN).

Figure 19 shows the displacement and axial force distribution trends of the steel anchor bolts after slope excavation. It can be seen that after slope excavation, the displacement and axial force of the anchor bolts increased rapidly, starting from the shotcrete pouring area and gradually decreased as the anchor bolts penetrate deeper into the slope.

**Figure 20:**
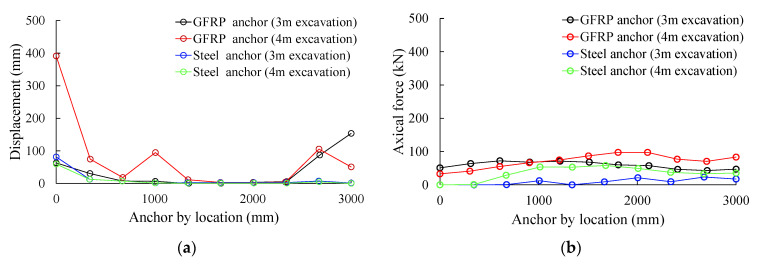
Axial force and displacement results generated from each strain sensor: (**a**) displacement; (**b**) axial force.

The displacement and axial stress distribution trends of the GFRP anchors were similar to those of the steel anchors. Table 5 lists the displacement and axial force results of the field tests and numerical analysis of the two anchors. A comparison of the displacement and axial force results for spirally reinforced steel anchors and GFRP anchors (Figure 20) revealed that the GFRP anchors exhibited higher displacement and axial force values than the spirally reinforced rock bolts. The rock bolt sensors were not anchored or prestressed during installation, and therefore were deemed to have no reinforcement effect. Individual element analysis results indicate that the displacement values of the rock bolts measured in the field were similar. Under the same conditions, the GFRP anchor sensors had superior displacement detection performance to those of spirally reinforced steel anchor sensors.

## 5. Conclusions

This study used GFRP anchor sensors and spiral steel anchor sensors to conduct slope collapse tests, analyzed the slope behavior characteristics at the initial stage of collapse, and explored the application potential of these two sensors in rock slope monitoring. The main conclusions obtained are as follows:

Field test results showed that shotcrete reinforcement significantly improved the shear strength of the slope and changed its deformation pattern. The reinforced slope exhibited “overall coordinated deformation” in the initial deformation phase, with anchor bolt displacement greater than that of the unreinforced slope, demonstrating effective support between the shotcrete layer and the rock mass. During the collapse phase, the unreinforced slope rapidly lost stability, while the reinforced slope only exhibited sustained plastic sliding, demonstrating a significant “deformation hysteresis effect”. This reinforcement method effectively slows the destabilization process, buying time for pre-disaster warning.

The GFRP anchors exhibited a shorter warning threshold arrival time, longer warning window, and higher and more stable strain sensitivity in different excavation stages. Their early warning capability was significantly better than that of the spiral steel anchors. Shotcrete could effectively improve the controllability of slope deformation by extending the slope instability time, and the reinforcement effect was more prominent in deep excavation scenarios. The coordinated application of the two can significantly improve the safety level of slope projects.

The results of the discrete element analysis show that because the anchor sensor was not anchored and prestressed during installation, it did not produce any reinforcement effect. The displacement and axial force values of the GFRP anchor were higher than those of the spiral steel anchor, which were similar to the results of the field measurements. Under the same conditions, the displacement detection performance of the GFRP anchor sensor was better than that of the spiral steel anchor sensor.

In current monitoring practices, this system can replace the traditional combination of spiral steel anchors and surface displacement monitoring. By leveraging the GFRP anchor’s ability to detect small deformations early and the shotcrete’s role in controlling overall deformation, a collaborative ‘deep displacement–surface reinforcement’ monitoring system can be established. This system is particularly suitable for slope projects in harsh environments such as rainy and corrosive environments. In terms of scalability, by adjusting the GFRP anchor length and optimizing the shotcrete mix, it can cover multiple scenarios, including mine spoil dumps and high highway slopes, providing a practical parameter standard and technical paradigm for existing intelligent slope early warning systems.

## Figures and Tables

**Table 1:** Mechanical parameters of the materials.

Material Parameters	Experimental Test Value
Internal friction angle (°)	36
Cohesion (kPa)	18
Poisson’s ratio (μ)	0.26
Water content (%)	7
Dry density (g/cm^3^)	2.25

**Table 2:** GFRP product specification.

Material Parameters	Values
Breaking load (kN)	25
Tensile strength (MPa)	850
Shear strength (MPa)	150
Elastic modulus (GPa)	60
Glass content (%)	75

**Table 3:** Early warning quantitative indicators.

Quantitative Indicators	GFRP Anchor	Steel Anchor
Ultimate strain	1.42% (14,200 με)	0.15% (1500 με)
Warning threshold (με)	4260	450
Strain sensitivity (με/mm)	28.5	23.9

Note: “Strain sensitivity” is defined as the strain change corresponding to unit displacement (ε/ΔS).

**Table 4:** Quantitative comparison table of anchor bolt warning performance.

Excavation Stage	Anchor Type	Time to First Warning Threshold (s)	Time to Instability (s)	Warning Time (s)	Corresponding Displacement (mm)	Strain Sensitivity (με/mm)
3 m	GFRP anchor	391	505	114	90	28.4
3 m	Steel anchor	404	493	89	13	24.0
4 m	GFRP anchor	4011	4059	48	397	28.5
4 m	Steel anchor	4016	4059	43	143	23.9

**Table 5:** Displacement and axial force results from the field experiments and numerical analysis.

Excavation Condition		Maximum Displacement (mm)		Maximum Axial Force (kN)
		3DEC	Field Test	3DEC
GFRP anchor	3 m	156.8	185	73.4
Steel anchor	3 m	59.8	31	23.4
GFRP anchor	4 m	394.2	308	98.7
Steel anchor	4 m	81.6	54	58.4

## Data Availability

Data will be made available on request.
